# A thermally baffled device for highly stabilized convective PCR

**DOI:** 10.1002/biot.201100453

**Published:** 2012-01-13

**Authors:** Hsiao-Fen Grace Chang, Yun-Long Tsai, Chuan-Fu Tsai, Ching-Ko Lin, Pei-Yu Lee, Ping-Hua Teng, Chen Su, Chien-Chung Jeng

**Affiliations:** 1 Department of Research and Development, GeneReach Biotechnology CorporationTaichung, Taiwan; 2 Institute of Medical Biotechnology, Central Taiwan University of Science and TechnologyTaichung, Taiwan; 3 Institute of Nanoscience & Department of Physics, National Chung Hsing UniversityTaichung, Taiwan

**Keywords:** Methods, Rayleigh-Bénard convective PCR, Thermally baffled PCR instrument, Yellow head virus

## Abstract

Rayleigh-Bénard convective PCR is a simple and effective design for amplification of DNA. Convective PCR is, however, extremely sensitive to environmental temperature fluctuations, especially when using small- diameter test tubes. Therefore, this method is inherently unstable with limited applications. Here, we present a convective PCR device that has been modified by adding thermal baffles. With this thermally baffled device the influence from fluctuations in environmental temperature were significantly reduced, even in a wind tunnel (1 m/s). The thermally baffled PCR instrument described here has the potential to be used as a low-cost, point-of-care device for PCR-based molecular diagnostics in the field.

## 1 Introduction

Polymerase chain reaction (PCR) is a molecular biology technique used for nucleic acid amplification [1]. PCR consists of three main steps: denaturation, annealing, and extension. The target DNA fragment is duplicated with the completion of one cycle of the three steps [2]. After 20–40 repeated cycles, the target DNA fragment is exponentially amplified and can be readily detected. With its high sensitivity and specificity, PCR has become a powerful tool for molecular diagnostics to detect the pathogens in plants, animals, or humans [3–7].

PCR is commonly carried out in a thermocycler, which repeatedly performs the heating and cooling of the reaction tube to achieve the required temperature for each step of the PCR. However, conventional thermocyclers are typically equipped with a hot-plate design with a metal block of high heat conductivity combined with plastic multi-well plates of low thermal conductivity, which severely hinders the achievable heating and cooling rate. Consequently, the majority of the time required is wasted on controlling the temperature changes of the instrument, rather than driving the PCR. In addition to the time needed to perform the reaction, the high cost of thermocyclers and the requirement of well-trained technicians also limit the application of PCR-based assays for point-of-care use, regardless of its high sensitivity and specificity.

Rayleigh-Bénard convection, which is driven by buoyancy when heating a fluid layer from below, is a common physical phenomenon. In 2002, Krishnan et al. [8] established the Rayleigh-Bénard convective PCR using a simple design that can generate a fluid density gradient to continuously circulate the reaction components through sequential temperature zones to carry out each stage of the PCR. Recently, Chou et al. [9] used a capillary tube as the chamber to carry out the Rayleigh-Bénard convection and PCR amplification. Since the buoyant driving force for the Rayleigh-Bénard convection came from the density difference in the upward and downward streams due to the temperature gradient, there was no need to repeatedly raise and lower the temperature manually, and therefore the device design could be much simpler than the conventional PCR apparatus. Moreover, in conventional PCR thermocyclers, increasing the ratio of the surface of the reaction tube in contact with the heater can improve the speed of temperature changes. In comparison, in the convective PCR, the heat transfer mainly takes place between the upstream and downstream of the convective flow, and does not dissipate into the environment [10]. Therefore, the process of heat transfer or utility rate of heat energy in convective PCR is more efficient than that of the conventional PCR. Thus, it only needs a heater with low power to create the convective cycle.

However, because the heat dissipation per unit volume by thermal conduction, which equals 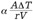, is almost proportional to 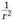 in a cylinder (where *r* is the distance from the stream center to the tube wall, *α* is thermal conductivity, *A* is the contact area, *V* is the volume, and Δ*T* is the temperature difference), it can affect the temperature gradient and cause fluctuation. This can disrupt the stability of the convective circulation loop [9, 10]. This occurs particularly easily in thin capillaries of a few millimeters diameter, making convective PCR inherently unstable and limiting its further applications. Consequently, though the principle of the convective PCR is simple and has been reported, no commercial product has been available so far. Therefore, it is critical to stabilize the temperature gradient inside the reaction tube to achieve successful convective PCR.

Here we report a convective PCR device that has been modified by adding a thermal baffle. Using this device we were able to minimize the impact of the environmental temperature fluctuation and carry out successfull PCR amplifications. Our results indicate that, with its simple design, the thermally baffled PCR instrument has the potential to become a powerful tool for point-of-care molecular diagnostics.

## 2 Materials and methods

### 2.1 Thermally baffled device

**Figure 1:**
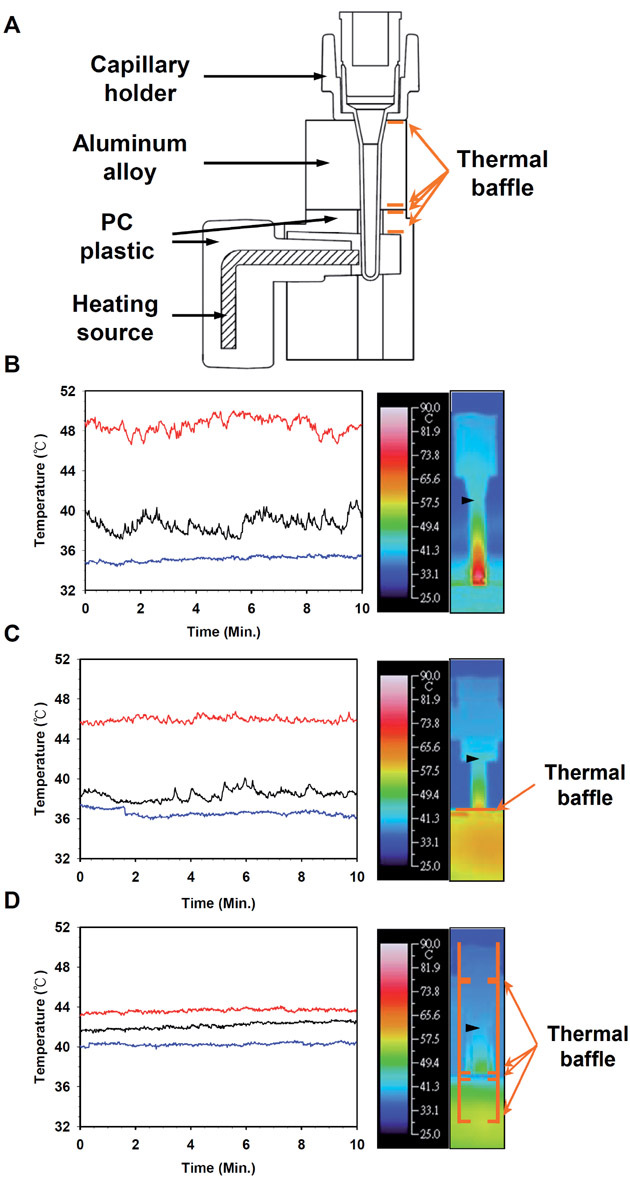
Design of the thermally baffled device and the temperature profile inside the capillary tube. (**A**) The thermally baffled device was designed to reduce the influence of the environment. (**B**) Thermo-image analysis showing the temperature profile with a bottom-heating apparatus only, (**C**) same bottom-heating apparatus covered with polycarbonate, or (**D**) a thermally baffled device. The thermo-image was observed using an infrared thermometer. The area of the thermal baffle is indicated by orange lines and arrows. The temperature was measured at 18.5 mm from the bottom of the tube (black arrowhead) and analyzed by a ThermoData Analysis System. The temperature profiles of the above three models under three different settings (28°C, black line; 38°C, red line; and 28°C with wind, blue line) were also compared. The temperature profile was obtained for three independent experiments with similar results.

The thermally baffled device was designed to reduce the environmental influence on the convection inside the capillary reaction tube. The heating source was covered with polycarbonate to reduce the heat radiation, and was designed to provide a constant heat of 95°C when the instrument started its program. The plastic capillary reaction tube was specially designed with a convection area of 20 mm in height with a height/diameter ratio of 11.6, and a Y shape opening for easy loading. To insulate the reaction tube from the external influence, aluminum alloy was used as a shield for compartmentalization and to baffle the vertical and horizontal air convection surrounding the tube (Fig. 1A).

### 2.2 Thermal image analysis

To determine the temperature variation of reagent inside the capillary reaction tube, an infrared thermometer (TVX-500EX, NEC Avio Infrared Technologies Co. Ltd.) was used to collect the temperature and the thermo-image. Twenty minutes after turning on the thermo-source, the temperature was measured each second at 18.5 mm from the bottom of the tube and recorded for 10 min. The temperature profile was then analyzed using the ThermoData Analysis System (NEC Avio Infrared Technologies).

### 2.3 PCR amplification and electrophoresis

The plasmid containing a sequence specific to Yellow head virus (YHV) was used as the template to amplify a specific 67-base-pair (bp) fragment. Forward (5'-TCGTCCCGGCAATTGTGAT-3') and reverse (5'-CCAGTGACGTTCGATGCAATA-3') primers specific to YHV were designed and used. The PCR mixture (50 μL), containing the target template, 0.5 μM forward primer, 0.5 μM reverse primer, 0.5 mM dNTP, 25 U Taq DNA polymerase (BioMi, Taichung, Taiwan), 50 mM Tris-HCl pH 8.3, 75 mM KCl, 3 mM MgCl_2_, and 1 mM DTT, was introduced into the capillary tubes (GeneReach, Taiwan) and incubated in the thermally baffled device for 30 min or the PCR thermocycler (ABI 2720, Life Technologies Corporation) using the program: denaturation at 95°C for 2 min, followed by 35 cycles of 95°C for 30 s, 60°C for 30 s, and 72°C for 30 s, and a cycle of 72°C for 30 s. The products were then analyzed by electrophoresis on a 12% polyacrylamide gel and visualized by ethidium bromide staining.

## 3 Results and discussion

Initially, a convective PCR device and related plastic capillary tubes were used to study the effect of environmental temperature fluctuation. Reverse transcription PCR buffer (50 μL; 50 mM Tris-HCl pH 8.3, 75 mM KCl, 3 mM MgCl_2_) was introduced into a capillary tube. The distance between the heating source and the top of buffer was 20 mm, and a constant heat of 95°C applied at the bottom. On placing the device in an open environment, and raising the surrounding temperature by 10°C, using thermo-imaging nearly the same amount of the temperature increase was observed at 18.5 mm from the bottom of the tube (Fig. 1B). When the device was placed inside a 28°C wind tunnel, the temperature dropped by 3.5°C. When the bottom-heating apparatus was insulated by a low-thermal-conductivity polycarbonate, the variation induced by the surrounding environment reduced slightly, showing the temperature fluctuation was mainly caused by the heat dissipation along the tube (Fig. 1C). Using the convective PCR device, we found that the liquid temperature inside the tube was very sensitive to the environment and the resulting temperature fluctuation could easily exceed the tolerable range for thermal convection and PCR. Moreover, it has been reported that even in a suitable environment, PCR in tubes with a low height/diameter ratio could be disrupted by the formation of multiple circulation cycles caused by the longitudinal instability [8, 10]. Therefore, it was critical to determine how a steady convection flow of the convective PCR device could be maintained to carry out PCRs properly.

To minimize the influence of the environment, a modified heat-baffled convective PCR device was designed. We covered the heating source with polycarbonate, and separated the exterior of the tube into compartments using aluminum alloy to baffle the vertical and horizontal air convection surrounding the tube (Figs. 1A and D). By controlling the contact area between the upper parts of the tube and the cooling fin, we stabilized the convection while preserving the heat. With the thermal baffle, we found that we were able to insulate the modified device with a single isothermal heat source from environmental influence, and stabilize the thermal convection flow inside the tube. As shown in Fig. 1D, with the thermal baffle, the temperature of the tube increased by only 1.5°C as the environment temperature increased by 10°C from 28°C, and the temperature fluctuation was reduced to 0.11–0.14°C. In addition, the temperature dropped by 1.8°C inside the wind tunnel. This implies that thermal baffle are able to significantly reduce the impact of the environmental temperature fluctuation and make the device suitable for PCR amplification.

**Figure 2:**
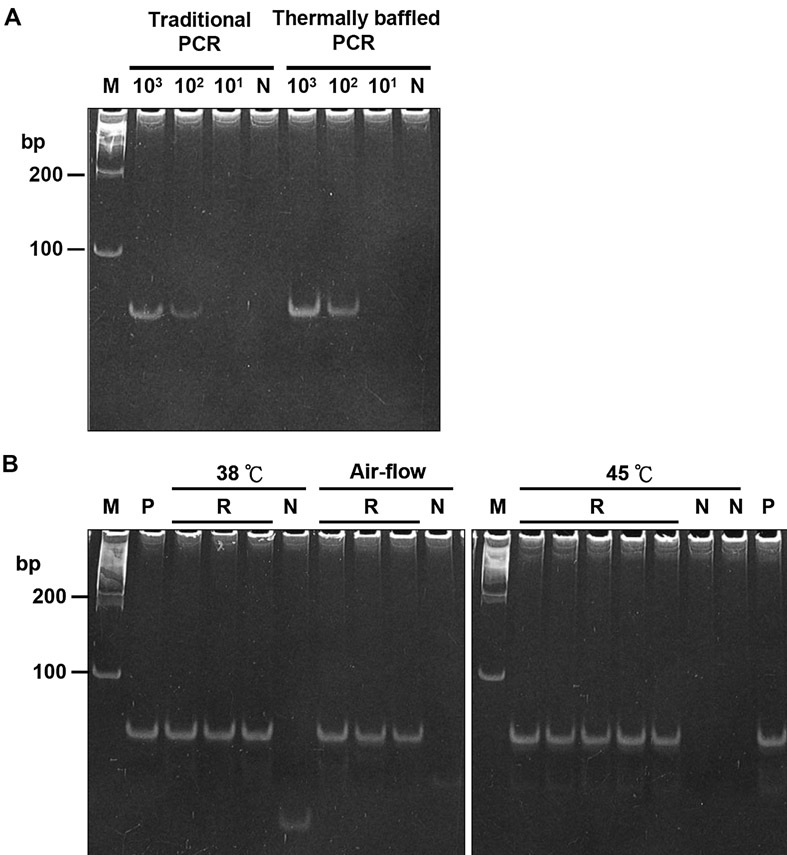
YHV-specific amplicon was amplified by conventional PCR and convective PCR using the thermally baffled device. (**A**) Different numbers of copies (10^3^–10^1^) of a plasmid containing a YHV-specific sequence as the template were amplified by conventional PCR or convective PCR with the thermally baffled device at 28°C, (**B**) 38°C, 45°C or under stable air-flow conditions (1 m/s). The products were analyzed on a 12% polyacrylamide gel in 1 × TAE buffer. M, DNA molecular weight marker (bp); R, reaction; N, no template control; P, positive control (at 28°C). PCR amplification was performed in at least three independent experiments with similar results.

To test PCR amplification using the thermally baffled device under different environmental temperature fluctuation from room temperature to 45°C (28°C, 38°C, or 45°C, respectively) or the windy conditions, we used a plasmid containing a sequence specific to YHV as the template to amplify a specific 67-bp fragment. The 50-μL PCR mixture was incubated in the thermally baffled device for 30 min, and the products were then analyzed by electrophoresis on a 12% polyacrylamide gel. For comparison, the same mixtures were subjected to conventional PCR amplification performed in a PCR thermocycler (ABI 2720, Life Technologies, Carlsbad, CA). The 67-bp fragment specific to YHV, which was confirmed by DNA sequencing analysis (data not shown), was successfully amplified by the thermally baffled device, and the sensitivity was as good as the conventional PCR with a sensitivity of 10^2^ copies per reaction (Fig. 2A). Our results show that PCR using the thermally baffled device, which provided insulation with a single isothermal heat source, can reach amplification efficiency comparable to that of conventional PCR. In addition, the YHV-specific amplicon could also be generated when the thermally baffled device was placed in an oven at 38° or 45°C, or under an air-flow condition (Fig. 2B), suggesting that the device can function in various environments.

The PCR amplification rate is (2^*α*^)^*t*/*τ*^ [11, 12], where α is the amplification efficiency and *τ* is the period of one PCR cycle. The period *τ* of the convective PCR (few seconds) is much shorter than that of a conventional PCR (2–3 min). The thermal baffle created an insulated environment, significantly reduced the influence of the environmental temperature fluctuations, and stabilized the thermal convection flow so that the PCR could be carried out successfully. Compared to the conventional PCR, for which a typical run takes about ∼2 h, the thermally baffled convective PCR takes only 10–20 min to achieve the desired results. Since only a single heat source is needed without complicated parts, the cost of a thermally baffled convective PCR device can be kept low. With its simple design and short turn-around time, the thermally baffled PCR instrument has significant potential to be used as a low-cost, point-of-care device for molecular diagnostics that many can afford.

## 4 Concluding remarks

Convective PCR is extremely sensitive to environmental temperature fluctuations, and therefore is inherently unstable with limited applications. In this study, we designed a thermally baffled convective PCR device and found that with the thermal baffle, the influence from the environmental temperature fluctuations was significantly reduced, and PCR amplification was able to be carried out successfully with a much shorter reaction time. With its simple design and short turn-around time, the thermally baffled PCR instrument can provide a powerful tool for point-of-care molecular diagnostics that many can afford.

## Abbreviation

YHV: Yellow head virus
